# The Value of Homatropine in Ophthalmic Practice

**Published:** 1881-02

**Authors:** F. C. Hotz

**Affiliations:** Chicago


ARTICLE II.

### THE VALUE OF HOMATROPINE IN OPHTHALMIC PRACTICE. By F. C. HOTZ, M.D., Chicago.

This new mydriatic, which was briefly noticed in the November number of the JOURNAL AND EXAMINER (p. 477), is destined to play a prominent part in ophthalmic practice. It seems to be just the remedy long wanted for the purpose of certain examinations of the eye, and to close an unpleasant gap in the small list of useful medicines for local applications upon the eye.

The form in which it is sold* and used, is the hydrobromate of homatropine. This salt forms small, transparent, needle-like crystals, which are readily dissolved in water. The solution is perfectly clear and colorless, and not liable to spoil. When dropped upon the conjunctiva of the lower lid, it produces some congestion of its blood vessels, and a peculiar, sometimes slightly smarting sensation. One gentleman compared the sensation to that created by very weak sulphuric acid upon the tongue. In every instance this slight irritation subsided spontaneously inside of fifteen minutes.

Its effect upon the eye is exactly that of a solution of sulphate of atropia—it dilates the pupil and suspends the power of accommodation by paralyzing the ciliary muscle. And still the two mydriatics show a remarkable difference in their action, inasmuch as the effect of homatropine subsides in twenty-four hours, while the effect of an equally strong solution of sulphate of atropia lasts many days.

---

* It can now be obtained of Messrs. Gale & Blocki, 85 Clark street, in this city.

When a drop of a one per cent. solution of atrop. sulph. (gr. v, ad. ℥j) is put in a healthy eye, the pupil shows the first sign of dilatation within fifteen minutes, and complete expansion in twenty or twenty-five minutes. The full mydriasis lasts about forty-two hours, but the pupil does not recover its normal size and mobility until the eleventh or twelfth day.

A solution of 1-18 per cent. (gr. j, ad. ℥iv) produces a good dilatation of the pupil in thirty minutes, and complete mydriasis in sixty minutes, and the effect of this weak solution even is visible till the seventh day.

In order to limit the effect to twenty-four hours, it is necessary to use a solution of the extreme attenuation of 1-144 per cent. (gr. j, ad. ℥xxx), which, however, produces only an incomplete mydriasis, after forty to sixty minutes,* and does not affect the accommodation in an appreciable degree.

Homatropine, on the other hand, manifests a tendency in the opposite direction; its effect is short lasting, and we can, it seems, scarcely make its solution strong enough to prolong its effect beyond twenty-four hours. A few observations, selected from a series of experiments with solutions of various strength, may serve to illustrate it.

1. *Observation with a five per cent. solution.*—When I first received a small quantity of homatropine, the solution was made five per cent. instead of one per cent.; but I did not discover the mistake until I had used it in the eye of a young lady for the purpose of paralyzing the accommodation. The lady, sixteen years of age, came to the city to be relieved of convergent strabismus of a high degree. The sight of each eye, tested before the operation, was found 20–30; it was made worse by convex spherical glasses, although the ophthalmoscope revealed a high degree of hypermetropia; nor was it benefited by convex cylindrical glasses, although the existence of astigmatismus was established by certain tests.

Four days after the tenotomy of both internal recti muscles, another examination of the sight had the same result. I then put one drop of the solution of homatropine, just received, in

* See Donders, "Anomalies of Refraction and Accommodation."

each eye. It smarted a little, and the conjunctiva became flushed. In ten minutes the pupils began to expand, in twenty minutes they were completely dilated; and in thirty minutes, when the sight was tested, the accommodation was completely suspended; each eye showed hypermetropic astigmatismus corrected by + 8 ⊂ + 36c, and V = $\frac{20}{20}$. I saw the patient again two days later; the pupils of her eyes were small, and readily responded to the influence of light; and she could read the finest type (Jäg. 1) with + 10 ⊂ + 36c, which combination suited her eyes best also for the distance.

2. *Observation with two per cent. solution.*—A boy, eleven years old, delicate and scrofulous, complained that when reading, his eyes often ached (they felt as if he "had sticks in them"), and the letters got blurred. There was considerable hyperæmia of the tarsal conjunctiva, with enlarged follicles in the lower retro-tarsal folds. V = $\frac{20}{20}$, worse with + 60; but he read Jäg. 1 no nearer than eight inches. Ophthalmoscope showed a normal fundus and H $\frac{1}{36}$. Homatropine was put in the eyes at 11 o'clock a. m., and began to enlarge the pupils at 11:10; at 11:20 complete dilatation and H $\frac{1}{50}$; at 11:30 H $\frac{1}{36}$; and at 12 o'clock also H $\frac{1}{36}$. I am informed that the pupils remained dilated until 6 p. m.; after that hour they slowly grew smaller, and recovered their normal size in the afternoon of the next day. In the morning of this day he could easily read ordinary print, and at noon the smallest print in the daily *Tribune.*

3. *Observation with one per cent. solution.*—On December 20, a young lady consulted me in regard to her eyes, which very often, in reading, get red, and smart, and overflow with tears. I found a marked degree of conjunctival hyperæmia, M $\frac{1}{50}$, V = $\frac{20}{20}$, and a normal condition of the fundus. For the purpose of ascertaining whether the slight myopia was real or due to spasm of the ciliary muscle, homatropine was used. At 11:20 a. m. two drops of a one per cent. solution were instilled in each eye; at 11:30, incipient dilatation of pupils; at 11:45, complete dilatation; at 11:50, accommodation paralyzed; cannot read ordinary print; M still $\frac{1}{50}$; at 12:30 p. m., M $\frac{1}{50}$, V = $\frac{20}{20}$; at 2:30, pupils still completely dilated; can read Jäg. 5 at eighteen inches, but no nearer; at 3:50 p. m., pupils showed a slight contraction; Jäg. 3 at seven inches; at 7 p. m., pupils perceptibly smaller; Jäg. 2 at seven inches. At noon of the following day pupils had recovered their normal size and activity.

4. *Observation with one-half per cent. solution.*—Dr. T. W. Campbell, at the Eye and Ear Infirmary, kindly allowed his left eye to be used for the experiment. Tested before the use of homatropine, this eye was found emmetropic, with near point at eight inches, $V = \frac{20}{20}$. The doctor's age is thirty-six years. The instillation of homatropine caused a flushing of the conjunctiva and a peculiar sensation which the doctor compared with the sensation very weak sulphuric acid produced upon the tongue. 2:25 p.m., homatropine applied in left eye; 2:35 p.m., left pupil a little larger than the right, but still responding to light; 2:45 p m., complete dilatation of pupil and paralysis of accommodation; $V = \frac{20}{20}$, emmetropia; could not read very large print (Jäg. 18), but read Jäg. 1 exactly at twelve inches with +12; if, however, the print was put half an inch nearer or further than twelve inches, he could not read it. At 4 p. m., the pupil showed the first indication of contraction, and the eye had recovered such accommodative power that it could see very large letters (Jäg. 19) at twenty inches; but after a few moments they became dim.

The doctor continued the reading tests, and furnished me with the following observations: At 5 p. m. he could read Jäg. 19; at 5:30 p. m., Jäg. 18; at 6:30 p. m., Jäg. 15; at 7:45 p. m., Jäg. 9; at 8 p. m., Jäg. 3; and on the afternoon of the next day, when I saw the doctor again, he could easily read Jäg. 1, although the left pupil was still a trifle larger than that of the right eye; the near point again at eight inches.

These observations, which substantially agree with the results of other experimenters, sufficiently illuminate the peculiar merits by which homatropine distinguishes itself from atropia. Both are equally prompt in dilating the pupil and in suspending the power of accommodation; but it requires almost two weeks for an eye to recover from the effect of a one per cent. solution of sulph. atrop., while the effect of a solution of homatropine of equal and even much greater strength subsides entirely within twenty-four hours. This rapid but brief effect of homatropine at once determines its relation to atropia in ophthalmic practice. It cannot be used advantageously (and consequently will not displace atropia) in the treatment of iritis and kindred diseases in which a continued uniform mydriasis is of paramount importance. And this is a very fortunate circumstance for the patients, inasmuch as homatropine is a rather expensive medicine—one grain costs about seventy cents at wholesale, so that the prescription druggist would charge at least two and a half or three dollars for one ounce of a one half per cent. solution (which is the standard solution of sulph. atrop. usually employed).

But homatropine will be employed in lieu of atrop. sulph. whenever we desire to dilate the pupil temporarily for the purpose of ophthalmoscopic examinations, or whenever we wish to suspend the accommodation in testing the eye for certain anomalies of refraction. For such purposes homatropine is the mydriatic *par excellence*, and possesses great advantages over atrop. sulph. It removes many annoyances which patients had to submit to in using atropia. In order to protect his eyes with dilated pupils against the unpleasant or even dangerous effect of bright daylight, the patient has to wear dark glasses or to remain in a darkened room until the natural mobility and size of the pupil is restored. With atrop. sulph. it required from seven to twelve days; with homatropine hardly as many hours.

It is a matter of no small consideration for a business man to be deprived of the full use of his eyes for nearly two weeks; and I have often noticed the great reluctance with which he consented to the use of atropia during the examination of his eyes when he was informed of the inconvenience necessarily resulting from it. I have known people to defer the examination on this account many weeks, or to give it up entirely.

In the case of a child, people dislike very much the idea that it will be unable to attend school for nearly two weeks after atropia is put in its eyes.

And a very serious question, involving a great loss of time or money, or both, may be the application of atrop. sulph., if the patient come to town from a distant city. If his eyes need glasses for astigmatism, and were tested under the influence of atropia, he was left in this dilemma: either he might go home, to return to the city after two weeks for the final examination, or he had to remain in the city two weeks, until the effect of the atrop. sulph. would have passed off, and his eyes would be ready for the final examination. This alternative was very disagreeable, but unavoidable—at least I adhere, in my practice, strictly to this rule: I do not order glasses for astigmatic eyes which have been examined under the influence of a mydriatic, without having had another examination after the effects of the medicine have entirely subsided. For the combination of glasses which corrects the astigmatism very nicely and accurately under the influence of atropia, very often needs some alteration to suit the eyes after the return of the accommodation. The first glasses may be theoretically correct; but the patient sees no satisfaction in knowing that, if he cannot use them. Suppose we have found a hypermetropic astigmatism corrected under atropine by $+ 8 \subset\!\supset + 36c$, but after the return of accommodation the patient rejects these glasses because they positively dim his sight, while he can see perfectly well with $+ 10 \subset\!\supset + 36c$. This patient would care very little to know that the first glasses are mathematically correct, as long as he cannot use them; he will consider the right glasses those which give him the clearest sight and the greatest comfort. For this reason I regard the final examination, after the activity of the pupil and ciliary muscle has returned, as necessary, if we wish to be certain that the prescribed glasses will give the patient the expected comfort.

This long delay of the final examination, together with other annoyances, is removed by substituting homatropine for atrop. sulph. In six hours the pupil has regained sufficient activity to render the eye all necessary protection against bright light; in twelve hours the accommodation has recovered such power that the patient can read and write tolerably well; and in forty-eight hours, at the latest, the final test in astigmatic eyes may be made, even when a strong solution of homatropine was used.

In the most instances a one-half per cent. solution will answer all purposes; it is strong enough to produce the desired enlargement of the pupil in fifteen or twenty minutes, and to paralyze the accommodation in thirty or forty minutes. Stronger solutions do not seem to take effect in a shorter time than fifteen or twenty minutes, and for this reason we gain nothing by their use under ordinary circumstances. Only in cases of hypermetropia of young persons, and in astigmatic eyes where we have reason to suspect the existence of habitual spastic contraction of the ciliary muscle, the use of a stronger (two per cent.) solution is indicated in order to insure a speedy and complete relaxation of the ciliary muscle.

It has been stated by Prof. Ladenburg, the discoverer of homatropine, that it is not poisonous. I consider my experience too limited yet to venture an opinion on this point; but if homatropine really is non-poisonous, and consequently its use is not likely to produce any constitutional disturbances like atropia and duboisia, it has an additional advantage over its rivals, and may justly be placed at the head of the list of mydriatics.

